# Electrostatic switch mechanisms of membrane protein trafficking and regulation

**DOI:** 10.1007/s12551-023-01166-2

**Published:** 2023-12-06

**Authors:** Ronald J. Clarke

**Affiliations:** 1 School of Chemistry, University of Sydney, Sydney, NSW 2006 Australia; 2 The University of Sydney Nano Institute, Sydney, NSW 2006 Australia

**Keywords:** Lipid-protein interaction, Intrinsically disordered proteins, Phosphatidylserine, Protein kinases, Lipid transverse asymmetry, Polybasic protein segments

## Abstract

Lipid-protein interactions are normally classified as either specific or general. Specific interactions refer to lipid binding to specific binding sites within a membrane protein, thereby modulating the protein’s thermal stability or kinetics. General interactions refer to indirect effects whereby lipids affect membrane proteins by modulating the membrane’s physical properties, e.g., its fluidity, thickness, or dipole potential. It is not widely recognized that there is a third distinct type of lipid-protein interaction. Intrinsically disordered N- or C-termini of membrane proteins can interact directly but nonspecifically with the surrounding membrane. Many peripheral membrane proteins are held to the cytoplasmic surface of the plasma membrane via a cooperative combination of two forces: hydrophobic anchoring and electrostatic attraction. An acyl chain, e.g., myristoyl, added post-translationally to one of the protein’s termini inserts itself into the lipid matrix and helps hold peripheral membrane proteins onto the membrane. Electrostatic attraction occurs between positively charged basic amino acid residues (lysine and arginine) on one of the protein’s terminal tails and negatively charged phospholipid head groups, such as phosphatidylserine. Phosphorylation of either serine or tyrosine residues on the terminal tails via regulatory protein kinases allows for an electrostatic switch mechanism to control trafficking of the protein. Kinase action reduces the positive charge on the protein’s tail, weakening the electrostatic attraction and releasing the protein from the membrane. A similar mechanism regulates many integral membrane proteins, but here only electrostatic interactions are involved, and the electrostatic switch modulates protein activity by altering the stabilities of different protein conformational states.

## Introduction

Lipids are a primary component of all biological membranes. The classical fluid-mosaic model of cell membranes postulated by Singer and Nicolson in 1972 (Singer and Nicolson 1972) represented the membrane as a fluid lipid bilayer matrix in which integral membrane proteins are embedded and to which peripheral membrane proteins can adsorb. The model that they presented displayed transverse asymmetry with respect to the proteins, i.e., some membrane proteins extend through the entire membrane whereas others only traverse part of the membrane and have surfaces exposed to either the cytoplasm or the extracellular medium. Singer and Nicolson’s model did not, however, include any asymmetry in the lipid composition of the membrane, neither lateral asymmetry in either plane of the membrane nor transverse asymmetry across the membrane. In this review, we will discuss mechanisms of trafficking and regulation of membrane proteins involving electrostatic interactions with charged lipids. These mechanisms are dependent on the transverse lipid asymmetry of the plasma membrane. Therefore, we begin with a discussion of the origin of this asymmetry. To put electrostatic switch mechanisms into the context of what is currently known about membranes and lipid-protein interactions, we will also touch on a number of other basic concepts of the physical chemistry of membranes and thus lay the foundation for the subsequent presentation of the idea of electrostatic switches.

**Fig. 1:**
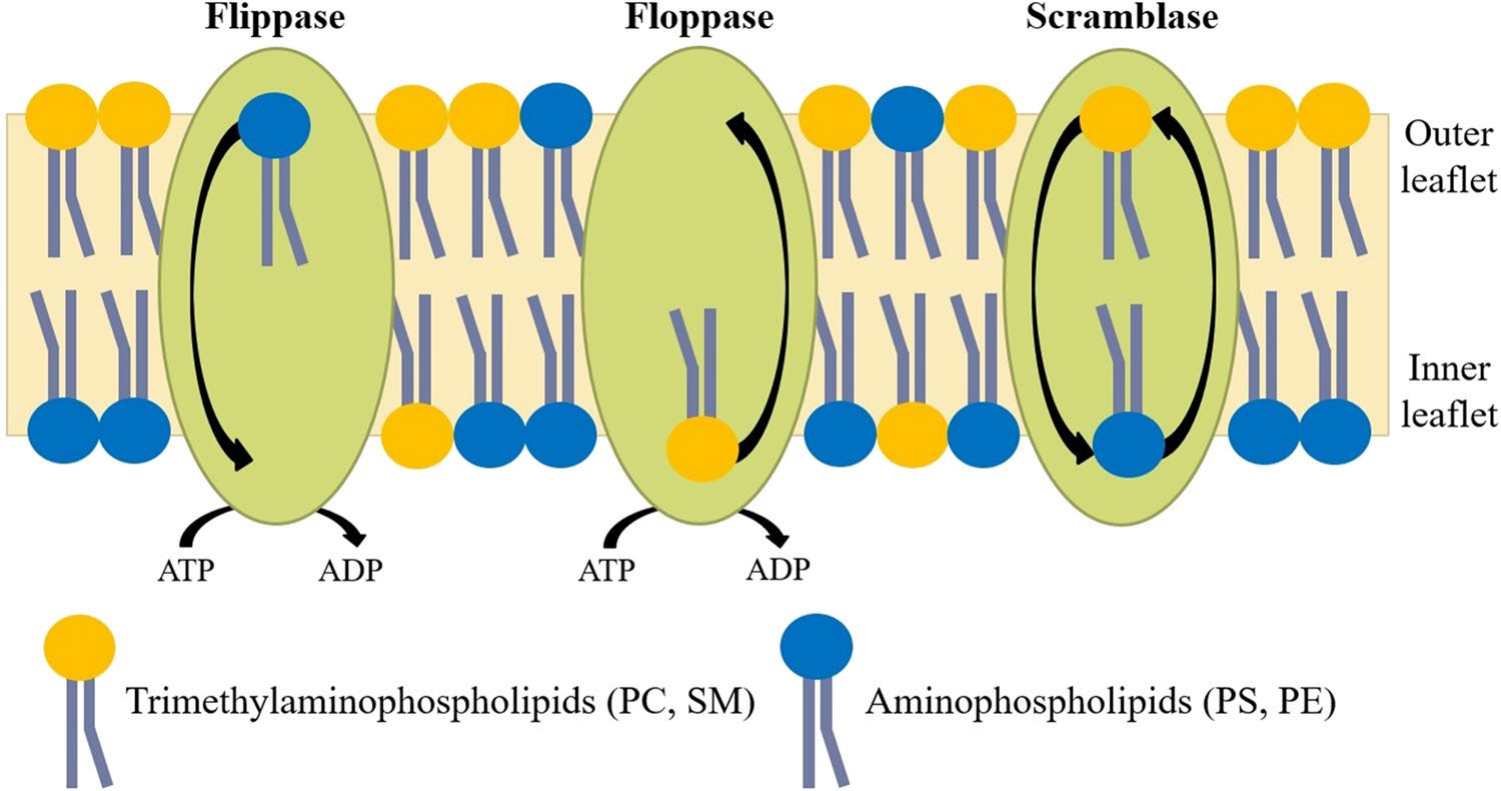
The three classes of phospholipid translocases (reproduced from Clarke et al (2020)). Most flippases transport aminophospholipids (PS and PE) from the outer to the inner leaflet of the plasma membrane. Floppases transport a variety of lipids (including PC) from the inner to the outer leaflet. Both flippases and floppases contribute to the maintenance of lipid asymmetry across the membrane, which is a nonequilibrium situation, and hence requires energy from ATP hydrolysis to drive the process. Scramblases transport phospholipids in both directions across the membrane, from the inner leaflet to the outer leaflet as well as from the outer to the inner leaflet, hence approaching an equilibrium distribution across the membrane and abolishing lipid asymmetry. Because this is a spontaneous process no energy is required, but scramblases require activation, e.g., by an increase in the cytoplasmic Ca^2+^ concentration or by peptide cleavage. The net directions of lipid transport by scramblases are identical to those of passive lipid flip-flop through the lipid phase of the membrane but facilitated or catalyzed by the scramblase protein

Although it did not receive as much attention as Singer and Nicolson’s paper, less than two weeks later a paper appeared by Bretscher (Bretscher 1972) providing experimental evidence using red blood cells that the lipid component of the membrane is also distributed asymmetrically across the membrane. Bretscher’s findings were subsequently found to be valid for most animal cells (Op den Kamp 1979; Zachowski and Devaux 1990; Devaux 1992; Zachowski 1993). The aminophospholipids, phosphatidylserine (PS) and phosphatidylethanolamine (PE), are located primarily in the cytoplasmic leaflet of animal plasma membranes, whereas the trimethylaminophospholipids, phosphatidylcholine (PC), and sphingomyelin (SM) are concentrated in the extracellular leaflet (Zachowski 1993; Clarke et al 2020). In living cells with membranes containing both lipid and protein, the movement of lipid molecules from one membrane leaflet to another (termed flip-flop) occurs on the minute timescale (Hirata and Axelrod 1978; de Kruiff et al. 1978; Nguyen et al 2019; Taylor et al 2019). Therefore, the only way that such a nonequilibrium asymmetric distribution of lipids across the membrane can be maintained is by the input of energy. The lipids must be actively pumped across the membrane. The first active transport of lipids, in this case PE and PS, utilizing the energy derived from the hydrolysis of ATP was discovered in 1984 by Seigneuret and Devaux (Seigneuret and Devaux 1984). Several years later, the same group was able to partially purify a PS-selective ATPase lipid pump from the red blood cell membrane (Morrot et al 1990). Cloning of the gene responsible for aminophospholipid translocation subsequently showed (Tang et al 1996) that the lipid transport was due to a P-type ATPase, i.e., the family of enzymes to which the ion-transporting Na^+^,K^+^-ATPase, and sarcoplasmic reticulum Ca^2+^-ATPase also belong. P-type ATPases responsible for the pumping of aminophospholipids (PS and PE) from the extracellular leaflet of the membrane into the cytoplasmic leaflet are referred to as *flippases* (see Fig. 1), a term originally coined by Bretscher (Bretscher 1974a, b). In contrast, active lipid transporters which pump trimethylaminophospholipids from the cytoplasmic leaflet of the membrane to the extracellular leaflet are termed *floppases* (see Fig. 1). These have been found (Tarling et al 2013; Quazi and Molday 2013; Neumann et al 2017) to belong to the family of ABC (ATP binding cassette) transporters, an unrelated family to that of the P-type ATPase flippases. This family is most widely known for conferring multi-drug resistance to cancer cells by pumping anti-cancer drugs out of the cell.

If animal cells are continually expending energy to maintain lipid asymmetry across the membrane, then it seems obvious that it must play some important role. The lipids cannot simply maintain a fluid environment to allow membrane proteins to function, as Singer and Nicolson’s fluid mosaic model would imply. Furthermore, why would one need a multitude of different lipid molecules with different headgroups and headgroup charges (negative, zwitterionic, or neutral), different hydrocarbon chain lengths, and different degrees of saturation. The lipids must be playing crucial physiological roles. Some of these roles have already been discovered. It is likely that many more await discovery. Let us first consider some of the roles of transverse lipid asymmetry.

Apart from flippases and floppases, i.e., active lipid transporters, passive lipid transporters also exist which facilitate the movement of lipids in both directions across the membrane. These are referred to as *scramblases* (see Fig. 1), because they abolish lipid asymmetry. Under most physiological conditions scramblases are in an inactive state, but they play an important signaling role in a variety of physiological processes. Scramblase activation leads to the exposure of PS on the extracellular surface of the cell (Hankins et al 2014; Bevers and Williamson 2016; Clarke et al 2020; Sakuragi and Nagata 2023). The negative charge of the PS molecule acts as a signal attracting macrophages, leading to apoptosis. In blood platelets, it attracts clotting factors and promotes clotting, and it has also been implicated in bone mineralization by promoting hydroxyapatite crystallization (Bevers and Williamson 2016; Clarke 2021).

In addition to transverse asymmetry, neither are lipids randomly dispersed in each individual leaflet of a membrane. Molecular dynamics simulations indicate that every integral membrane protein requires its own unique local lipid environment (Tieleman et al 2021). Even in synthetic lipid vesicles or liposomes, domain formation can occur when lipid species are mixed, particularly when cholesterol is added. For example, vesicles formed using a single pure PC undergo phase transitions as the temperature is gradually increased; from a gel or solid-ordered, *S*_*o*_, phase with van der Waals forces between the hydrocarbon tails of the lipids in an all-*trans* conformation, through a so-called ripple phase and finally to the liquid crystalline or liquid-disordered, *L*_*d*_, phase where the van der Waals forces are disrupted and the hydrocarbon tails fluctuate between *trans* and *gauche* conformations. Simultaneously with the breaking of van der Waals forces between the tails, differential scanning calorimetric (DSC) experiments at different ionic strengths have shown that electrostatic interactions on the membrane surface are also broken (Garcia et al 2019). These interactions are most likely due to a hydrogen-bonded network between hydrating water molecules and polar lipid head-groups across the membrane surface (Chen et al 2010; Mondal et al 2012; Niyonyanagi et al 2013; Cyran et al 2018; Garcia et al 2019). However, once the cholesterol composition is increased to above ~ 30 mol%, i.e., into a typical physiological range for an animal plasma membrane, the phase transitions disappear and the membrane converts to the liquid-ordered, *L*_*o*_, phase at all temperatures from 0 to 100 °C, with a membrane fluidity intermediate between that of the gel and liquid crystalline phases (Mainali et al 2013; Subczynski et al 2017; Garcia et al 2019). At cholesterol levels below ~ 30 mol%, as the phase transitions are gradually disappearing, i.e., heat capacity peaks in DSC measurements are getting smaller, it appears very likely that separate domains of either *S*_*o*_ or ripple phase, which still undergo phase transitions, coexist with *L*_*o*_ phase which does not undergo a transition.

There is, however, a decisive difference between lateral domain formation by lipids and the transverse lipid asymmetry observed in living plasma membranes, discussed earlier. Lateral domain formation is a spontaneous process requiring no input of energy. It arises because of the different strengths of intermolecular forces between two or more lipid species and the effects of temperature on these forces. In contrast, transverse lipid asymmetry is not spontaneous and requires the constant input of energy in the form of ATP for it to be maintained. Although visualization has been a long-standing problem, coexisting *L*_*o*_ and *L*_*d*_ domains are likely to also exist in the plasma membranes of animal cells, again with cholesterol being a crucial component to form the *L*_*o*_ phase, and with certain integral membrane proteins preferring to colocalize in the *L*_o_ domains, others in the *L*_*d*_ domains and some at the *L*_*o*_/*L*_*d*_ phase boundaries. This is one definition of the so-called “lipid raft” hypothesis (Simons and van Meer 1988; Simons and Ikonen 1997; Kaiser et al 2009; Kusumi et al 2020; Suzuki and Kusumi 2023). The local phase behavior of the membrane and its effect on membrane fluidity would certainly be contributing factors in modulating the activity of membrane proteins. However, this is not the emphasis of this review and readers are referred to more comprehensive recent reviews on this topic (Kusumi et al 2020; Suzuki and Kusumi 2023). Here, we will focus on electrostatic mechanisms of lipid-protein interactions, with other types of lipid effects only described briefly as a comparison and to put the electrostatic effects into perspective.

## Specific lipid-protein interactions due to lipid binding within a membrane protein

Interactions between lipids and membrane proteins are generally classified as either general or specific (Hossain and Clarke 2019), although, as we shall soon see, this classification can be somewhat blurred. Specific lipid interactions are usually attributed to lipids that co-crystallize with the membrane protein concerned and are located at specific sites within the protein’s three-dimensional structure. Many examples of this exist. For example, in the case of the Na^+^,K^+^-ATPase or sodium pump, three individual lipid binding sites have been identified in published structures (Kanai et al 2013; Habeck et al 2015, 2017). Phospholipids have also been found to bind within the three-dimensional structure of the gastric H^+^, K^+^-ATPase (Abe et al 2018), and the sarcoplasmic reticulum Ca^2+^-ATPase or SERCA (Drachmann et al 2014). The proteins taken as examples here are integral membrane proteins belonging to the P-type ATPase family, i.e., they are all ion pumps which are continually undergoing conformational changes within the membrane as they pump ions against an electrochemical potential gradient across the membrane. The lipids which bind to the specific sites within the protein, if they have any effect at all, could have three possible effects on the proteins. They could stabilize the protein against thermal degradation, they could enhance the activity of the protein, i.e., increase its ion pumping rate, or they could inhibit the protein’s activity. All three of these effects have been observed experimentally (Habeck et al 2015, 2017). Lipids which increase thermal stabilization presumably fill clefts within the protein structure, thus increasing the rigidity of the protein and reducing conformational flexibility. Effects on ion pumping activity must be due to either energetic stabilization or destabilization of a protein conformational state either side of a rate-determining step in the ion pump’s catalytic cycle. Thus, if a reactant state is stabilized by interaction with a specific lipid relative to the product state, one would see a decrease in activity due to an increase in the activation energy of the reaction step. Conversely, if a reactant state is destabilized by interaction with a specific lipid relative to the product state, one would see an increase in activity due to a decrease in the activation energy of the reaction step.

## General lipid-protein interactions due to a change in membrane physical properties

**Fig. 2:**
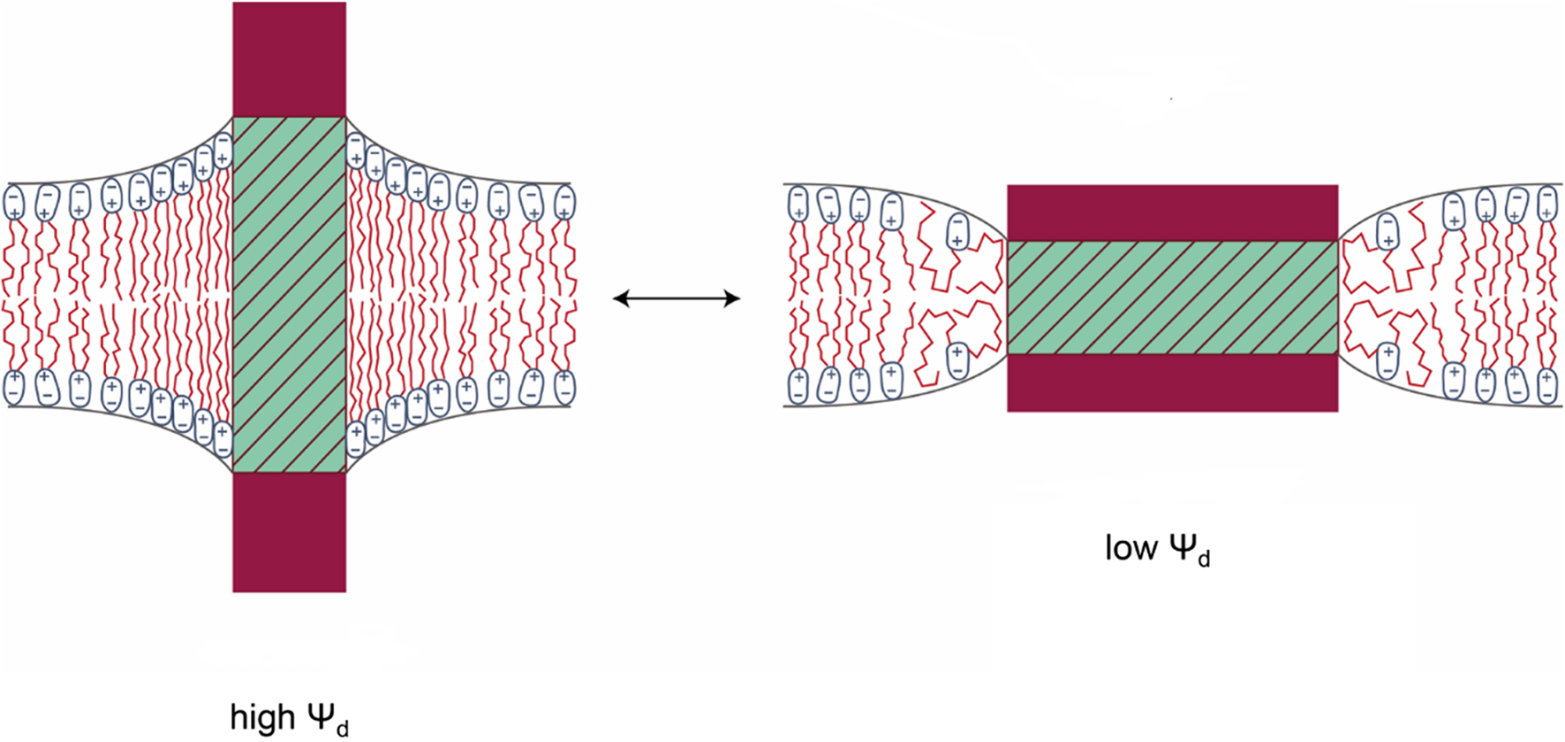
Hydrophobic matching between a membrane protein and its surrounding lipid membrane (reproduced from Clarke (2015)). Prevention of hydrophobic mismatch between the protein and the membrane could require local bending of the membrane. This causes local changes in the membrane dipole potential, *ψ*_*d*_, in the lipid packing density, in the strength of van der Waals forces between the lipid tails and in their entropy. The figure represents an integral membrane protein (e.g., an ion pump) undergoing a large conformational change which drastically changes its hydrophobic thickness. Based on Le Châtelier’s principle, the high *ψ*_*d*_ state would be stabilized by any membrane additive that decreases *ψ*_*d*_ and the low *ψ*_*d*_ state would be stabilized by any membrane additive that increases *ψ*_*d*_

When one speaks of general lipid-protein interactions, what is normally meant are effects of lipids in the lipid matrix surrounding a membrane protein, with the lipids exerting their effect on the protein via a change in one or more physical properties of the membrane. For example, lipids can have different hydrocarbon chain lengths, which influences the hydrophobic thickness of the membrane and how well it matches the hydrophobic thickness (see Fig. 2) of a transmembrane protein (Mouritsen and Bloom 1984, 1993; Fattal and Ben-Shaul 1993; Clarke 2015). The hydrocarbon tails can also have different degrees of saturation, which influences the fluidity of the membrane and hence the freedom with which a membrane protein can undergo conformational changes. Increasing degrees of unsaturation also decreases the packing density of the lipids, which decreases the membrane dipole potential, *ψ*_*d*_ (Clarke 1997; Peterson et al 2002; Warshaviak et al 2011), i.e., the electrical potential difference located within lipid membranes in the narrow region between the glycerol backbone of a phospholipid and the interface with the neighboring aqueous solution (Brockman 1994; Clarke 2001; O’Shea 2003; Wang 2012). Another factor influencing the magnitude of *ψ*_*d*_ is the type of linkage between the hydrocarbon tails and the glycerol backbone, i.e., whether it is an ester or ether linkage, with the carbonyl group of ester linkages causing a higher *ψ*_*d*_ (Gawrisch et al 1992; Clarke 1997) than ether linkages. Further contributing factors to the magnitude of *ψ*_*d*_ are the nature of the lipid head group and the number and type of hydrocarbon chain, with *sn*-2 chains causing a higher *ψ*_*d*_ than *sn*-1 (Starke-Peterkovic and Clarke 2009). The value of *ψ*_*d*_ is normally in the range 100–500 mV, depending on the membrane lipid composition. Because it drops over a small distance, it produces large electric field strengths of 10^8^–10^9^ V m^−1^.

If a transmembrane protein undergoes a conformational transition, e.g., associated with ion occlusion in the case of an ion pump or a gating transition in the case of an ion channel, which changes the hydrophobic thickness of the membrane, the surrounding lipid membrane must deform to avoid hydrophobic mismatch between the protein and the membrane (see Fig. 2). The change in lipid packing that this deformation or bending causes must result in a change in the free energy of the combined system, i.e., protein plus surrounding membrane. On the membrane side, there are several contributions. If the membrane bends out to accommodate a protein with a larger hydrophobic thickness (see Fig. 2, left), the lipid tails must stretch out to increase the membrane thickness. This increases the packing density of the lipids, leading to increased electrostatic repulsion in the headgroup region because the dipoles responsible for *ψ*_*d*_ are now closer together. However, the higher packing density increases the strength of van der Waals attractions between the lipid tails. Simultaneously, there is a decrease in entropy of the tails, which is unfavorable in terms of free energy. If the membrane needs to bend in to accommodate a protein with a smaller hydrophobic thickness (see Fig. 2, right), then the situation is reversed. Electrostatic repulsion in the headgroup region is reduced due to the decreased packing density necessary to thin the membrane, van der Waals forces between the tails are weakened, and the entropy of the tails is increased. For a real plasma membrane containing asymmetrical membrane proteins, bending out may require a different amount of energy as bending in, but both must require energy because the membrane geometry deviates from its spontaneous lowest energy state of a flat surface. Nevertheless, bending is necessary, because otherwise hydrophobic regions of the protein would be exposed to the surrounding aqueous environment, which would be an even greater energy cost.

The relative energies of the two states shown in Fig. 2 are dependent on the membrane lipid composition and on the addition of any molecules that bind to the membrane. The fundamental equilibrium principle of Le Châtelier states that equilibria always shift to minimize any perturbation. Thus, if any substance is incorporated into the membrane that increases *ψ*_*d*_, the equilibrium in Fig. 2 must shift to the low-*ψ*_*d*_ state, i.e., to the right, to the product state, which would stimulate the activity of a membrane protein. Conversely, if any substance is incorporated into the membrane that decreases *ψ*_*d*_, the equilibrium must shift to the high-*ψ*_*d*_ state, i.e., to the left, to the reactant state, which could inhibit the activity of a membrane protein. Here we have focused on *ψ*_*d*_. However, the same principles hold for shifts in equilibria of membrane proteins for membrane additives that change any other membrane physical property. The membrane protein equilibrium will always shift to decrease whatever change in membrane physical property the additive has caused.

An interesting alternative to the scheme shown in Fig. 2 was discovered for the sarcoplasmic reticulum Ca^2+^-ATPase (SERCA) (Norimatsu et al 2017). Rather than the membrane bending to accommodate a changed hydrophobic thickness of the protein when it undergoes a conformational change, Norimatsu et al. (2017) found that the transmembrane helices of SERCA tilt, so that its hydrophobic domains still fit within the membrane. Thus, membrane bending or protein tilting are both mechanisms whereby hydrophobic matching can be achieved when an integral membrane protein undergoes a conformational change.

## Specific lipid-protein interactions with lipids in the lipid matrix

Earlier, we discussed specific interactions of membrane proteins with lipids embedded within the protein. However, a topic that is increasingly attracting attention is segments of proteins that embed themselves in or adsorb to the lipid matrix. Many membrane proteins possess intrinsically disordered extensions on their N- or C-termini that can interact directly with the lipid membrane either electrostatically or via the hydrophobic effect (Cornish et al 2020). Intrinsically disordered proteins and intrinsically disordered domains of proteins are currently an active field of research. It has become clear that the one sequence-one structure dogma for which Christian Anfinsen (Anfinsen et al 1961) won the 1972 Nobel Prize in Chemistry needs revision (Cornish et al 2020; Clarke 2020). It is true that the amino acid sequence of a protein can code for a particular three-dimensional structure, but not always. An amino acid sequence can also code for disorder. In fact, disordered sequences, because of their ability to change their structure, are ideally suited to the purposes of signaling and protein regulation. This has been recognized since the early 1990s for peripheral membrane proteins but is only recently being recognized to be true also for integral membrane proteins. Because these are two very distinct types of membrane proteins, we will discuss them separately.

## Peripheral membrane proteins and reduction of dimensionality

Peripheral membrane proteins are proteins that bind transiently to the surface of a membrane, in contrast to integral membrane proteins, which are embedded in the membrane. A prime example is the Src kinase, a tyrosine kinase that interacts with several integral membrane proteins and phosphorylates tyrosine residues (Sigal et al 1994). A good question to ask would be, why does it need to be a peripheral membrane protein at all? Why doesn’t it just stay in the cytoplasm and diffuse through the cytoplasm to whichever tyrosine needs to be phosphorylated? The key to this is the concept of reduction of dimensionality, first treated mathematically for biological diffusion processes by Adam and Delbrück (Adam and Delbrück 1968). By binding to the membrane surface, the Src kinase can diffuse across the membrane surface until it finds its target tyrosine, which is also located on the membrane, and avoids getting lost in the cytoplasm. Thus, the dimensionality of the diffusion is reduced from 3 to 2. This can significantly increase the chances of the Src kinase finding its target and, thus, increase its catalytic rate. The situation is analogous to that of heterogeneous catalysis, of which a prime example is the Haber–Bosch process, the main industrial process for producing ammonia (NH_3_) and for which three Nobel Prizes in Chemistry have been awarded: Haber (1919), Bosch and Bergius (1931), and Ertl (2007). In this reaction nitrogen gas, N_2_, and hydrogen gas, H_2_, bind weakly to a solid metallic surface and are split into their constituent atoms which then diffuse across the surface to one another, where they can meet, recombine, and form ammonia (Ertl 2008). Avoiding three dimensions and reducing the diffusion to two dimensions across the surface dramatically enhances the rate of reaction.

Binding to a surface, a membrane in the case of the Src kinase, is, however, not the only requirement to observe an acceleration in the reaction occurring. The binding must be sufficiently weak that the molecule or enzyme concerned can continually unbind and rebind at a fast rate to allow diffusion across the surface. If the binding is too strong, then the molecule would stay fixed in one position, so that the chance of it reaching its target would be reduced and one could even observe a lower rate of reaction than if the molecule stayed in the bulk phase and underwent three-dimensional diffusion. Therefore, it is important to consider how peripheral membrane proteins bind to the lipid membrane. But first, another way to consider the advantage of reducing the dimensionality is to simply consider the effect that it has on the local concentration of the peripheral protein next to the membrane. McLaughlin and Aderem (McLaughlin and Aderem 1995) estimated the factor by which the effective concentration of the protein is increased from the ratio of the volume of a spherical cell divided by the volume of a thin spherical shell adjacent to the membrane, i.e., (4/3)π*r*^3^/4π*r*^2^*d* = *r*/3*d*, where *r* is the radius of the cell and *d* is the thickness of the shell. For a cell of radius *r* = 10 μm and a shell of thickness *d* = 1 nm, the factor becomes 1,000. Thus, the apparent concentration of the protein next to the membrane could be increased a thousand-fold, which in the case of Src would greatly increase the probability of it phosphorylating a tyrosine residue of an integral membrane protein. It should be mentioned, however, that this calculation is a simplification, because the cytoplasm of a cell is a crowded environment, in which the presence of many macromolecules reduces the available free volume (Rivas and Minton 2016, 2022). This could increase further the probability of interaction of a peripheral membrane protein with the cytoplasmic surface of the plasma membrane.

## Peripheral membrane protein binding to the membrane surface

The forces that keep a peripheral protein adsorbed to the surface of the membrane must be in a delicate balance. They must be weak enough so that the protein can diffuse across the membrane, but they must be strong enough to keep the protein close to the membrane surface. Furthermore, a mechanism must exist for weakening the force of attraction to the membrane when necessary, to allow the protein to diffuse through the cytoplasm to another target membrane, e.g., the nuclear membrane. Two forces are responsible: the hydrophobic effect and electrostatic attraction.

**Fig. 3:**
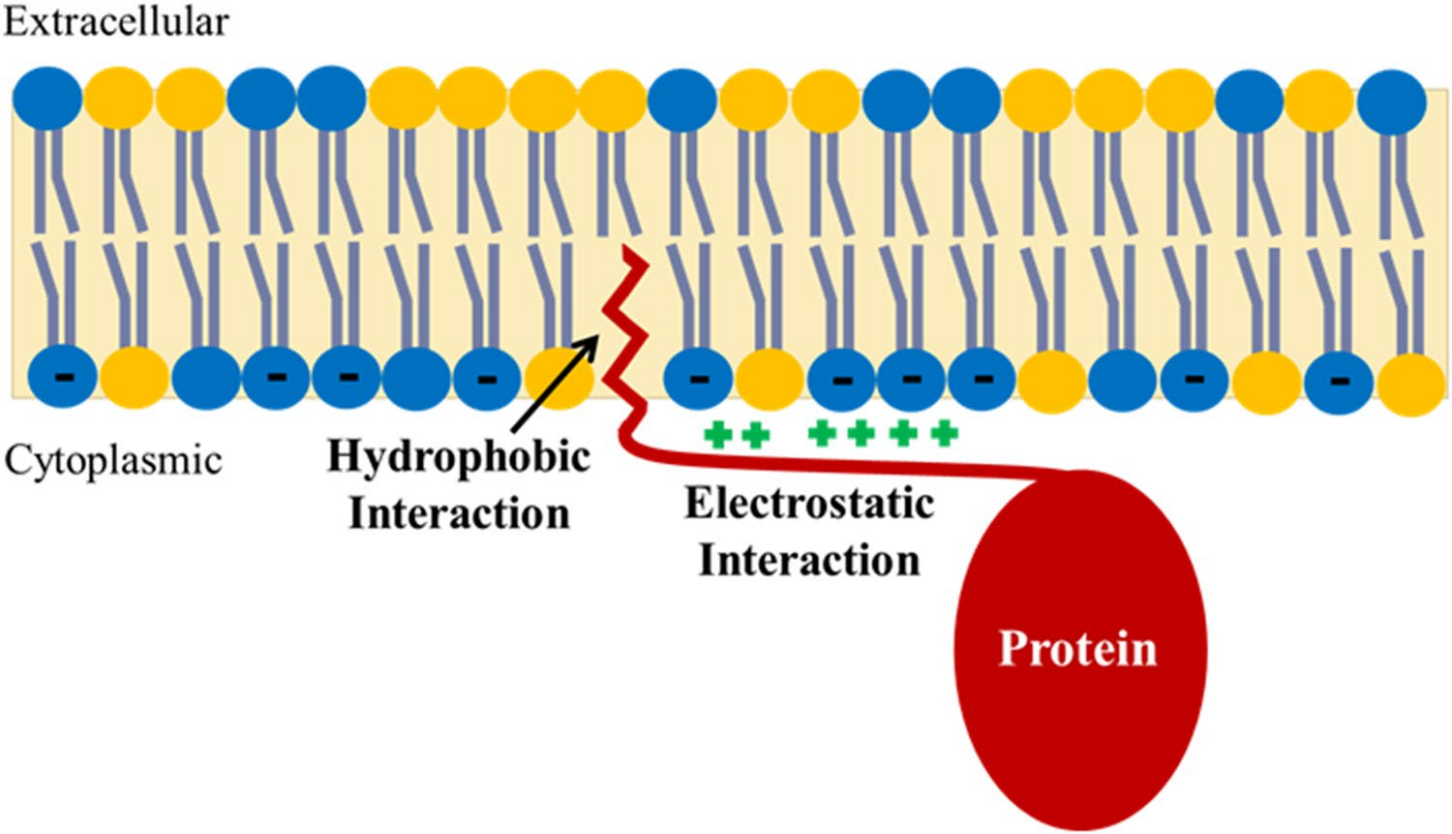
Membrane anchoring of peripheral membrane proteins (reproduced from Clarke et al 2020). Many peripheral membrane proteins have two thermodynamic contributions to their membrane anchoring: (1) a hydrophobic interaction arising from the insertion of a hydrocarbon acyl chain into the lipid bilayer and (2) an electrostatic interaction between positively charged basic amino acid residues of the protein (lysine or arginine) and negatively charged anionic lipid headgroups (predominantly phosphatidylserine) on the cytoplasmic face of the membrane. Membrane anchoring thus relies in part on the asymmetric transverse distribution of PS across the membrane, which is maintained by a flippase

Many peripheral membrane proteins undergo a posttranslational addition of an acyl chain, most commonly a myristoyl chain (C14:0) to either a glycine or cysteine residue at their N-terminus (Towler et al 1988; McLaughlin and Aderem 1995; Giglione and Meinnel 2022). Because of the free energy cost of exposing the hydrophobic myristoyl chain to the aqueous environment, it buries itself in the hydrocarbon interior of the lipid membrane (see Fig. 3). Membrane binding is favored by the release of low entropy water molecules around the hydrocarbon myristoyl chain to the bulk solvent and van der Waals forces between the myristoyl chain and those of the lipids within the membrane. Myristoyl thus acts as a hydrophobic anchor, helping to hold the peripheral protein to the membrane. McLaughlin and Aderem (McLaughlin and Aderem 1995) estimated an association constant of the myristoyl chain to a membrane on the order of 10^4^ M^−1^, corresponding to a dissociation constant of 0.1 mM. This interaction alone is not sufficiently strong to hold the peripheral protein onto the membrane. Therefore, many utilize a further trick.

As explained in the Introduction, the cytoplasmic surface of animal plasma membranes is negatively charged because of the continual lipid pumping activity of flippases, which maintains a concentration of the negatively charged lipid phosphatidylserine in the membrane cytoplasmic leaflet (see Fig. 1). To further strengthen their interaction with the membrane, peripheral proteins incorporate polybasic regions, i.e., containing lysine and arginine residues, in their intrinsically disordered N-termini. Because these basic amino acid residues are positively charged at neutral pH, this allows for an electrostatic attraction between the protein’s N-terminus and the membrane. The strength of the electrostatic attraction will depend on the number of basic residues and how localized they are along the polypeptide chain. However, McLaughlin and Aderem (McLaughlin and Aderem 1995) estimated an association constant in the order of 10^3^ M^−1^, corresponding to a dissociation constant of 1 mM. This is also too weak by itself to hold the protein onto the membrane. However, if the N-terminus has both a myristoyl addition and a polybasic region, as many do, then the hydrophobic anchor and the electrostatic attraction work cooperatively to hold the protein onto the membrane. The net association constant can then be estimated by multiplying the individual association constants for the two interactions, i.e., *K* = 10^4^ × 10^3^ = 10^7^, equivalent to a dissociation constant of 100 nM. Based on measurements with myristoylated and non-myristoylated peptides corresponding to the N-terminus of the Src kinase with both negatively-charged and neutral synthetic lipid vesicles, Buser et al. (Buser et al 1994) have shown that a dissociation constant of this magnitude is sufficient to bind the N-terminus to the cytoplasmic surface of a plasma membrane. The situation is analogous to the chelate effect in inorganic chemistry. A prime example is the hexadentate ligand EDTA^4−^ that binds strongly the Ca^2+^ ions. If one of its six bonds to the central ion is broken, there are still five holding it in place and the broken bond simply reforms. So it is with the N-myristoylated tail of a peripheral membrane protein. If either the hydrophobic anchor is released from the membrane or the electrostatic attraction breaks, then the protein’s tail will still not be released from the membrane, because the unbroken interaction will ensure that the broken one re-forms.

One further important point is that both these interactions are nonspecific. The myristoyl chain will bind equally well wherever it inserts into a membrane. Similarly, the negative charges of phosphatidylserine are located everywhere across the cytoplasmic surface of the plasma membrane. Therefore, an N-myristoylated tail containing polybasic regions will also bind equally strongly anywhere across the cytoplasmic surface of the membrane. This allows the protein’s tail and thus the entire peripheral membrane protein to stay close to the membrane but, on occasions when the hydrophobic and electrostatic interactions do simultaneously break, still be able to diffuse across the membrane surface in a two-dimensional fashion to find its substrate.

Although myristoyl is the most common acyl chain appended to the peripheral membrane proteins, it is not the only one. Other post-translational modifications include palmitoylation (i.e., addition of a 16-carbon chain) and prenylation, which involves addition of the poly-unsaturated and branch-chained farnesyl (total 15 carbons) or geranylgeranyl (total 20 carbons) to the protein (McLaughlin and Aderem 1995; Araya et al 2022). Why the addition of the 14-carbon myristoyl chain is, however, the most prevalent hydrophobic modification is an interesting question. It has been speculated (Towler et al 1988; McLauglin and Aderem 1995) that the smaller hydrophobic surface of the shorter myristoyl chain provides a weaker binding and hence more flexibility and reversibility in membrane binding. The smaller hydrophobic surface results in the release of less low entropy water to the bulk when it binds to the membrane, thus reducing the driving force for membrane binding.

## Electrostatic switch mechanism of peripheral membrane protein trafficking

**Fig. 4:**
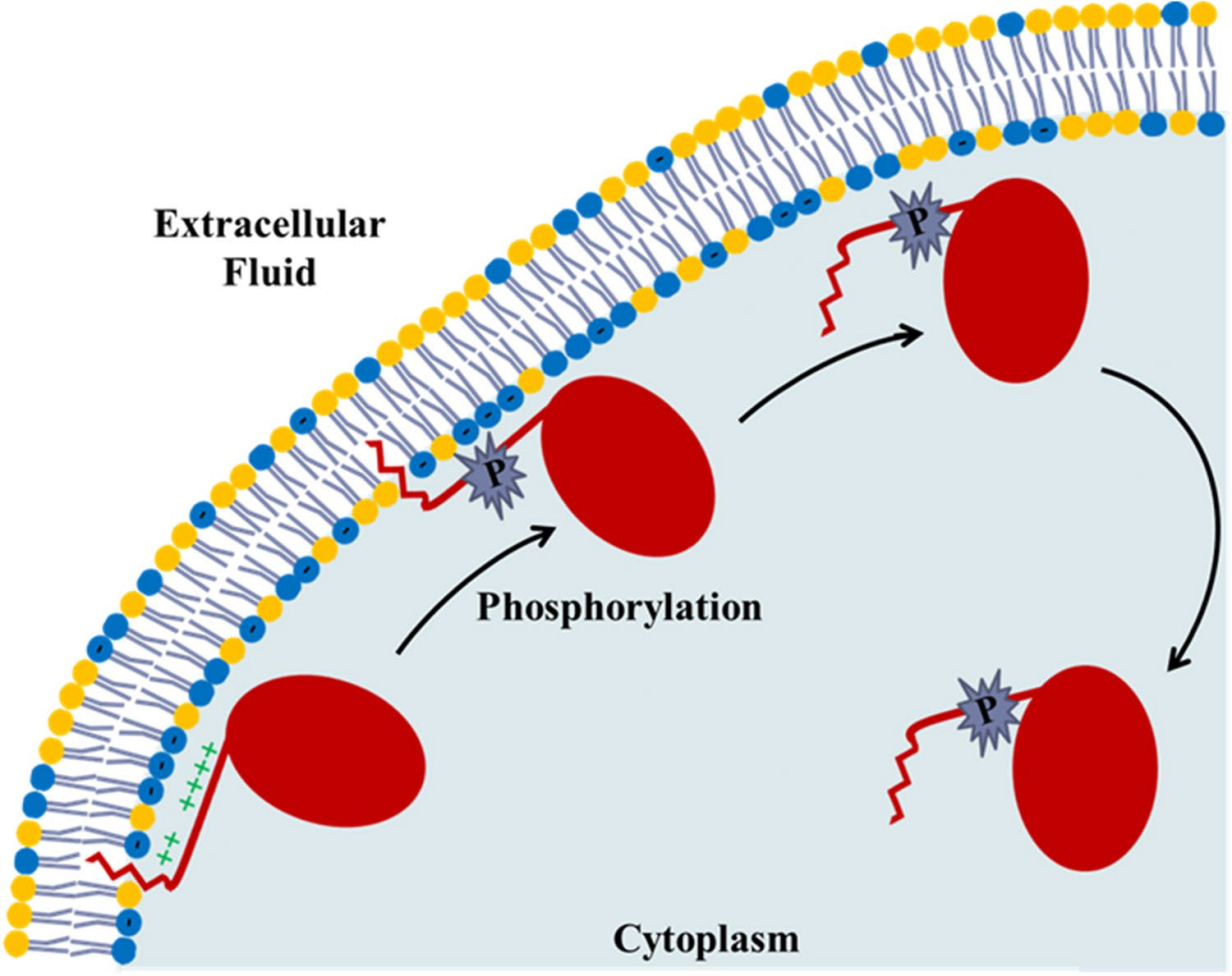
Electrostatic switch mechanism of peripheral membrane protein trafficking (reproduced from Clarke et al 2020). The left side of the figure shows a peripheral membrane protein anchored to the cytoplasmic membrane surface by hydrophobic and electrostatic interactions of its N- or C-terminus. Phosphorylation of a serine or tyrosine located within the protein’s positively charged polybasic region of clustered lysine and arginine residues by a protein kinase introduces negative charge, thus weakening the electrostatic attraction for the membrane. The hydrophobic interaction is not strong enough to hold the protein onto the membrane and it, therefore, dissociates and moves away from the membrane into the cytoplasm. Dephosphorylation of the serine or tyrosine by a phosphatase would remove the negatively charged phosphate and allow the protein to re-associate with the cytoplasmic membrane surface

Now it is important to consider not only how peripheral membrane proteins bind to the plasma membrane but also how they get off the membrane. Many of these peripheral proteins are enzymes that do not just act on the surface of the plasma membrane. They may also act in other locations of the cell, on the membranes of cell organelles. Therefore, when they are required elsewhere, they need to dissociate themselves from the membrane. So how is their trafficking within the cell controlled? This occurs via an electrostatic switch mechanism (McLaughlin and Aderem 1995; Yeung et al 2008; Maures et al 2011; Clarke et al 2020). As well as having polybasic regions of positive charge on their N- or C-terminal tails, these proteins also have serine and/or tyrosine residues which are targets for protein kinases. Once these residues become phosphorylated, this introduces negative charge onto the proteins’ membrane-binding tail, partially neutralizing the positive charges of the basic residues and weakening the electrostatic attraction to the membrane. The hydrophobic myristoyl anchor is by itself not strong enough to hold the tail onto the membrane and so the entire peripheral protein dissociates from the membrane (see Fig. 4). If the protein is required to bind to another membrane in another cell location, of course, the phosphate group introduced by the kinase needs to be removed again. This requires the action of a regulatory phosphatase (Giglione and Meinnel 2022). The exposure of the myristoyl chain to the aqueous environment of the cytoplasm, as shown in the final product state of Fig. 4, is certainly energetically unfavorable and an interesting question would be how this protein state can persist until the protein reaches another membrane. Although this is still unclear, a possibility might be that the myristoyl chain interacts with other hydrophobic domains in the protein’s interior.

Other versions of the electrostatic mechanism involve, rather than reducing the positive charge on the N- or C-terminal tail of the protein, reducing the negative charge on the membrane. One way this could happen is if Ca^2+^ ions are released from intracellular stores. Ca^2+^ can then bind to the PS headgroup, neutralizing its charge (Yeung et al 2008). Another way of reducing the membrane charge is activation of a scramblase. This would allow PS to redistribute across the membrane, so that it becomes exposed on the extracellular surface of the plasma membrane. This is what happens when a cell is undergoing apoptosis. The negative charges of PS on the extracellular surface are a signal for macrophages to engulf and dispose of the cell (Fadok et al 1992; Bevers et al 1998; Miyanishi et al 2007; Park et al 2009; Leventis and Grinstein 2010; Suzuki et al 2013; Hankins et al 2014; Birge et al 2016; Clarke et al 2020; Sakuragi and Nagata 2023).

The earliest known example of a peripheral membrane protein whose interaction with the plasma membrane is controlled by an electrostatic switch mechanism appears to be the myristoylated alanine-rich c-kinase substrate (MARCKS). MARCKS and MARCKS-like proteins are known to be essential for post-natal survival of vertebrates (Stumpo et al 1995). They crosslink actin filaments and have been shown to be involved in a variety of early cell developmental processes (El Amri et al 2018). In the late 1980s and early 1990s, the South African-born biologist and anti-apartheid campaigner Alan Aderem (then at Rockefeller University, New York) carried out cell biological measurements indicating that MARCKS is released from the plasma membrane by phosphorylation by protein kinase C (Wang et al 1989; Rosen et al 1990; Thelen et al 1991; Allen and Aderem 1995). Subsequent biophysical and biochemical studies (Kim et al 1994a, 1994b; Taniguchi and Manenti 1993; Peitzsch and McLaughlin 1993) utilizing model membrane systems of different lipid composition and synthetic peptides as models of the MARCKS protein provided a deeper understanding of the fundamental chemical interactions responsible for the binding and release of MARCKS from the membrane, leading to the concept of the electrostatic switch (McLaughlin and Aderem 1995; Aderem 1995; Seykora et al. 1996; Taniguchi 1999; Murray et al 2002).

As explained earlier, the major anionic lipid in the cytoplasmic leaflet of the animal plasma membrane is PS, which is concentrated in the cytoplasmic leaflet by the continual pumping action of flippases. Because the electrostatic interaction between polybasic domains of peripheral proteins and anionic lipids is nonspecific, one would expect PS to be the major binding partner of MARCKS. However, animal membranes also contain smaller amounts of negatively charged phosphatidylinositols (PI), which could also interact electrostatically with polybasic protein domains. Phosphatidylinositol 4,5-bisphosphate (PIP_2_) has an important signaling role in the cell, being the origin of two second-messengers: diacylglycerol (DAG) and inositol 1,4,5-triphosphate (IP_3_). Apart from interacting with actin when in the cytoplasm, it has also been suggested that a function of membrane-bound MARCKS could be to sequester and concentrate PIP_2_ into localized domains with the membrane (McLaughlin et al 2002). There is also growing evidence that actin of the cytoskeleton may also interact with both peripheral and integral membrane proteins at the membrane, possibly via unstructured N- or C-termini, thus providing a possible cytoskeleton-linked regulation of membrane proteins (Vasilev et al 2021).

Another peripheral protein that was postulated early to utilize an electrostatic switch mechanism is Src, a nonreceptor protein tyrosine kinase, which requires membrane binding for its function (Brown and Cooper 1996; Resh 1999). As in the case of MARCKS, experiments utilizing peptides mimicking the N-terminus of Src, membranes with varying lipid composition and charge, and theoretical calculations confirmed nonspecific electrostatic binding of Src to the cytoplasmic surface of animal membranes, in addition to a myristoyl hydrophobic anchor (Sigal et al 1994; Buser et al 1994; Murray et al 1998). Evidence also exists that its binding to the plasma membrane is modulated by phosphorylation, consistent with an electrostatic switch mechanism (Walker et al 1993; Pérez et al 2009), although Murray et al. (1998) only observed a small shift of a Src chimeric protein away from the plasma membrane to the cytoplasm on phosphorylation.

A number of other peripheral membrane proteins have been identified which possess both a hydrophobic anchor on either their N- or C-termini together with a polybasic cluster and whose cell location is likely to be controlled by an electrostatic switch mechanism: the Gag protein of retroviruses such as HIV-1 (Lei et al 2013), G proteins (Noguera-Salvà et al 2017), the SH2B1β gene product (Maures et al 2011), the GTPases K-ras4B, Rap1-A, Rac-1 and Rho-1 (Magee and Marshall 1999), and the BASP-1 protein (Murray et al 2002). The basic clusters of these proteins always contain positively-charged lysine residues (K), sometimes together with positively-charged arginine (R) residues (Murray et al 2002). The preference for lysine over arginine may be related to the strength of interaction with membranes and their degree of membrane penetration. Theoretical calculations show that an arginine residue penetrates more deeply into a phospholipid membrane than lysine (Li et al 2013). This could be explained by the delocalization of the arginine residue’s positive charge over its guanidinium group, whereas the ammonium group of lysine has a localized positive charge. Charge delocalization would decrease the Born- or self-energy of the arginine side chain relative to that of lysine, allowing it to penetrate further into the low dielectric medium of the membrane interior. Lysine, on the other hand, would be expected to stay on the membrane surface, where in biological membranes it could interact with anionic lipids such as PS. The shallower penetration of lysine into the membrane could then allow polybasic lysine clusters to more easily move on and off the membrane, thus facilitating the movement of peripheral membrane proteins between different cell locations.

## Electrostatic switch mechanism of integral membrane protein regulation

The relevance of electrostatic switch mechanisms for integral membrane proteins is less obvious than for peripheral membrane proteins, but it is now emerging that they also play important roles for integral membrane proteins. Of course, they have no need for a hydrophobic anchor because they have transmembrane domains holding them permanently embedded within the membrane. However, many of them possess disordered N- or C-terminal tails with membrane-binding polybasic clusters. For proteins that remain embedded in a membrane, an electrostatic switch mechanism can have no purpose in releasing them from the membrane to move to another location. Any electrostatic switch must, therefore, play another role. Although much research still needs to be carried out, the most likely explanation would seem to be that electrostatic switches of integral membrane proteins play a regulatory role, modulating protein activity. This is a more subtle but no less important role than that played by electrostatic switches for peripheral proteins. However, because electrostatic switches do not release integral membrane proteins from the membrane, it makes their discovery more difficult. This requires detailed investigation of the proteins’ activities and even measurement of individual reaction steps in an integral membrane protein’s complete reaction cycle.

### P-type ATPases

One family of integral membrane proteins for which it seems very likely that an electrostatic switch mechanism is operative is the P-type ATPase family, in particular, the Na^+^,K^+^-ATPase (or sodium pump) and the H^+^,K^+^-ATPase (or gastric protein pump). The Na^+^,K^+^-ATPase is present in the plasma membrane of every cell of every multicellular animal. One of its most fundamental roles is to maintain cell volume (Kay and Blaustein 2019). By continually utilizing the energy derived from the hydrolysis of ATP in pumping Na^+^ ions out of the cell cytoplasm into the extracellular medium, the Na^+^,K^+^-ATPase reduces the osmotic pressure inside the cell and prevents cell swelling. The enzyme thus maintains constant gradients of Na^+^ and K^+^ concentration and electrochemical potential across the membrane. It can be compared to a bilge pump of a boat, constantly pumping out incoming water to keep the boat on a constant level and not sinking. The Na^+^,K^+^-ATPase does not, however, simply expend the energy of ATP hydrolysis; in actual fact by doing the work of pumping Na^+^ ions across the plasma membrane, it converts and stores the energy in the form of a Na^+^ gradient across the membrane. This energy can then be utilized by numerous Na^+^-coupled secondary transporters for the transport of metabolites across the membrane (Kaplan 2002; Clarke and Fan 2011; Clausen et al. 2017). A prime example is the Na^+^, glucose cotransporter, which in the kidney utilizes the Na^+^ gradient to reabsorb glucose into the bloodstream. A certain amount of the energy of ATP hydrolysis is also released by the Na^+^,K^+^-ATPase in the form of heat. This is not wasted but helps to maintain body temperature (Clarke et al 2013). The H^+^,K^+^-ATPase, on the other hand, is the enzyme responsible for the acidification of the stomach, which is necessary for the activation of pepsin, a stomach enzyme that breaks down the protein of ingested food. After each bite of food, H^+^,K^+^-ATPase molecules are recruited to the plasma membrane of the parietal cells of the gastric mucosa, where they utilize the energy of ATP hydrolysis to pump H^+^ or protons into the stomach (Dunbar and Caplan 2001). The H^+^,K^+^-ATPase is the target of proton pump inhibitors, one of the world’s most widely prescribed class of drugs, used for the treatment of peptic ulcers and the prevention of stomach cancer (Sachs et al 2014).

Both the Na^+^,K^+^-ATPase and the H^+^,K^+^-ATPase possess polybasic clusters in the cytoplasmic N-termini of their catalytic alpha-subunits (Diaz and Clarke 2018). As in the case of the peripheral membrane proteins already discussed, these clusters consist predominantly of lysine residues. In addition, in the case of the Na^+^,K^+^-ATPase, conserved tyrosine and serine residues are present in all vertebrate species. These are prime targets for a regulatory Src kinase and protein kinase C (PKC), respectively. The N-terminus of the alpha-subunit of the H^+^,K^+^-ATPase also contains tyrosine and serine residues, but they are not as broadly conserved as those of the Na^+^,K^+^-ATPase. A tyrosine residue is conserved in all vertebrate species except cartilaginous fish. Another tyrosine residue is present in most mammals, but it is replaced by histidine in some. A serine residue is conserved in all placental mammals. This suggests that regulation by Src kinase and protein kinase C may also be occurring in the H^+^,K^+^-ATPase, but the regulation is somewhat more species-specific than for the Na^+^,K^+^-ATPase. Bioinformatic analyses of the amino acid sequences of the Na^+^,K^+^-ATPase and different isoforms of the PKC and Src kinase family to look for evidence of co-evolution as an indicator of a functional interaction have suggested that the θ and η isoforms of PKC are the most likely interaction partners with the conserved serine of the Na^+^,K^+^-ATPase N-terminus, and that Src itself is the most likely interacting kinase of the Src kinase family (Blayney et al 2023).

**Fig. 5:**
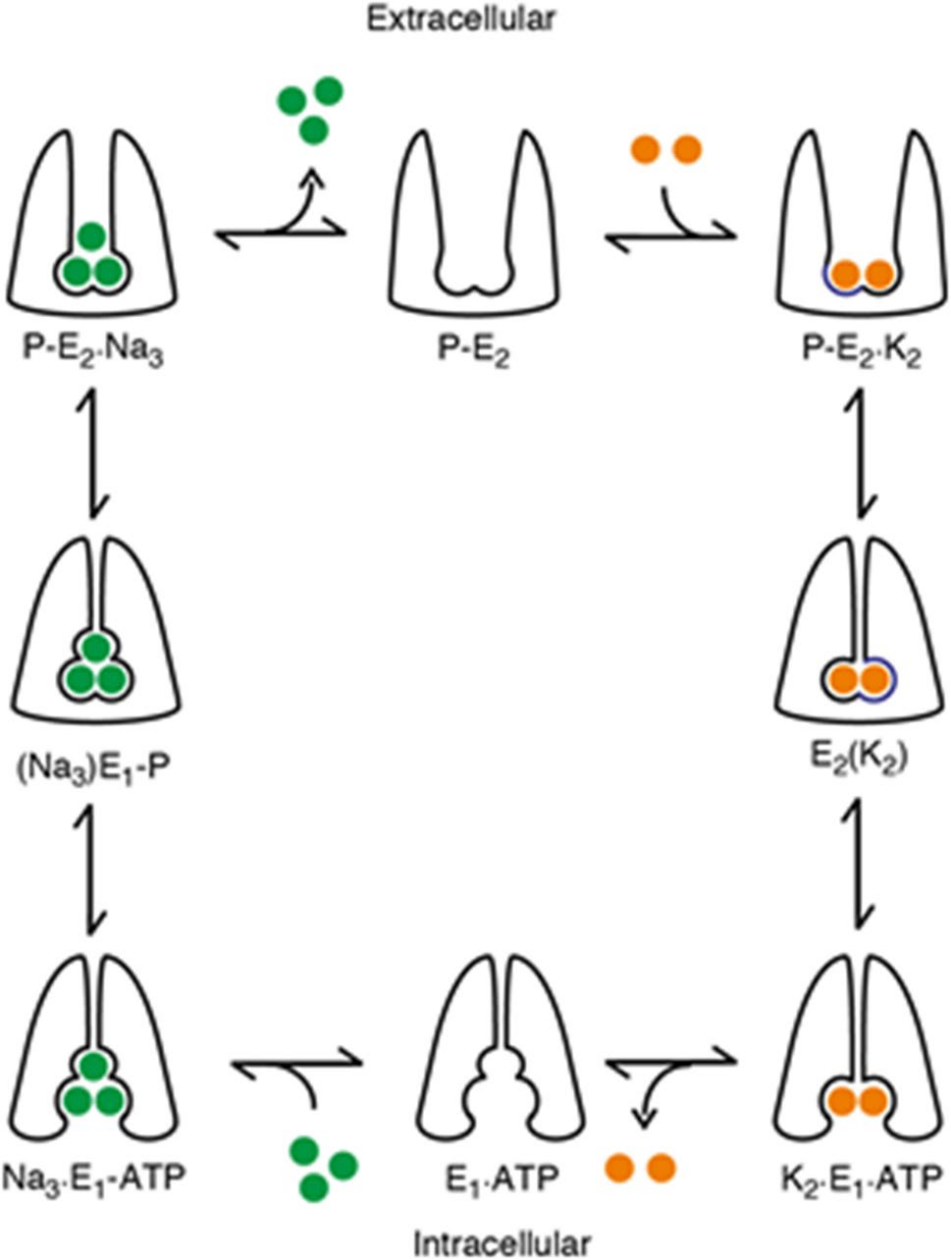
Schematic diagram of the ion pumping reaction cycle of the Na^+^,K^+^-ATPase or sodium pump (adapted from Gadsby et al 2012). Green and orange balls represent Na^+^ and K^+^ ions. Under physiological conditions, the pump proceeds in a clockwise direction around the cycle, taking up 3 Na^+^ ions from the cytoplasm and releasing them to the extracellular medium and taking up 2 K^+^ ions from the extracellular medium and releasing them to the cytoplasm for the hydrolysis of 1 ATP molecule. All other small ion-transporting P-type ATPases operate similarly, but with variations in the ions transported and the stoichiometry. ATP and Mg^2+^ binding and ADP and P_i_ release reactions are not explicitly included

Both experimental measurements and theoretical calculations using peptides corresponding to the amino acid sequences of the N-termini of both the Na^+^,K^+^-ATPase and the H^+^,K^+^-ATPase have indicated a membrane interaction involving anionic phospholipids such as PS (Nguyen et al 2018; Hossain et al 2021). Furthermore, the interaction is weakened on increasing the ionic strength, indicating an electrostatic origin. These experiments and the results obtained are very reminiscent of studies on the N-terminus of the peripheral MARCKS protein, already described (Kim et al 1994a, 1994b; Taniguchi and Manenti 1993; Peitzsch and McLaughlin 1993). Experiments on the purified Na^+^,K^+^-ATPase at varying ionic strength showed that breaking of an electrostatic interaction causes a shift of the enzyme from its K^+^-selective E2 conformation to its Na^+^-selective E1 conformation (see Fig. 5) (Jiang et al 2017). Analysis of the ionic strength dependence of the shift via the Gouy-Chapman theory and comparison with electrophoretic measurements of the charge density of the Na^+^,K^+^-ATPase-containing membrane fragments were found to be consistent with a change in interaction of the protein with the membrane surface as the origin of the conformational shift (Lev et al 2023).

Further experimental data supporting an involvement of the N-terminus in controlling the enzyme’s distribution between the E1 and E2 states were found from experiments in which the N-terminus of the Na^+^,K^+^-ATPase was cleaved using trypsin (Hossain et al 2020). Apart from their different affinities for Na^+^ and K^+^, the E1 and E2 conformations have very different affinities for ATP. ATP binds to the E1 state with a dissociation constant in the range 0.1–14 μM, with the value depending on the protein’s degree of oligomerization in the membrane (Pilotelle-Bunner et al 2008). In the E2 conformation ATP binding is much weaker, with a dissociation constant in the range 71–450 μM (Clarke 2009). Therefore, the change in ATP affinity can be used as an indication of the protein’s conformational state. For fluorescence measurements, it has been found that the dye eosin is a useful substitute for ATP (Skou and Esmann 1981, 1983) because it binds to the protein in the same location as ATP and undergoes a shift in its excitation spectrum as well as a change in fluorescence intensity, allowing easy detection. Cleavage of the N-terminus of the Na^+^,K^+^-ATPase was found (Hossain et al 2020) to significantly reduce the protein’s affinity for eosin when in the E1 state, shifting it towards the value found in the E2 state. This indicates that the N-terminus plays an important part in determining the high ATP affinity of the E1 state. Removal of the N-terminus either by proteolytic cleavage (Cornelius et al 2005; Jørgensen and Collins 1986) or by mutagenesis (Scanzano et al 2007) has also been found to cause a shift in the protein’s conformational equilibrium towards E1.

Involvement of the Na^+^,K^+^-ATPase N-terminus in the E2-E1 transition was also shown by Jørgensen and coworkers (Jørgensen and Collins 1986; Jørgensen et al 1982; Jørgensen and Andersen 1988; Jørgensen 1975) from the time course of proteolytic digestion experiments. In the E1 state, trypsin cleaves rapidly at Lys-30 on the N-terminus and subsequently at Arg-262, whereas in the E2 state, it cleaves first at Arg-438 and afterward at Lys-30. This suggests that, in the E2 state, Lys-30 of the α-subunit is initially protected from trypsin attack. Based on what we now know, it appears likely that this is due to interaction with the surrounding membrane. Jørgensen et al (1982) also found that trypsinolysis of the bond 30–31 of the N-terminus is strongly dependent on ionic strength, which is also consistent with an electrostatic interaction.

Unfortunately, it seems that around the middle of the first decade of the twenty-first century the N-terminus lost the focus of most researchers within the P-type ATPase field. This could have been due to the appearance of the first crystal structures of the Na^+^,K^+^-ATPase and an increase in attention on the ion transport sites, which could be visualized in the new structures (Morth et al 2007; Shinoda et al 2009; Kanai et al 2013; Nyblom et al 2013). In contrast, the N-terminus, because of its flexibility and intrinsically disordered structure (Diaz and Clarke 2018), could not be resolved and, therefore, the crystal structures provided no information on its role. Obviously, if a section of the protein cannot be directly observed, many scientists in the field tend to ignore it. In the case of the H^+^,K^+^-ATPase, the N-terminus was even removed prior to crystallization (Abe et al 2018). Recent structural determinations of the Na^+^,K^+^-ATPase by cryo-electron-microscopy (Nguyen et al 2022; Guo et al 2022) have, however, provided some information on the cytoplasmic ion gating mechanism of the protein which is relevant to the N-terminus. Although the N-terminus was still not resolved, the new structures showed that cytoplasmic gating involved significant movement of the first transmembrane helix. Because this helix is directly covalently linked to the N-terminus, it seems certain that cytoplasmic gating must also involve significant movement of the N-terminus. In fact, this was already proposed by Jørgensen and coworkers based on biochemical data alone in the 1970s and 1980s (Jørgensen 1975; Jørgensen et al 1982; Jørgensen and Collins 1986; Jørgensen and Andersen 1988). In recent years, it is increasingly becoming clear that static crystal structures do not tell the whole story. Intrinsically disordered proteins are now an active area of research. It is now recognized that the flexibility of disordered sequences allows them to interact more easily with regulatory partner enzymes, such as protein kinases, and this makes them ideally suited for regulatory and signaling processes (Wright and Dyson 2002; Dyson and Wright 2002; Burger et al 2014; Iakoucheva et al 2004).

Although the N-terminus could not be resolved in any of the published crystal structures of the Na^+^,K^+^-ATPase, an approach that has been used to obtain a complete structure is to predict the secondary structure of the N-terminus based on its amino acid sequence and then to tag it onto a published structure. After inserting the complete protein into a membrane, this then allows molecular dynamics simulations to be run to investigate the mobility of the N-terminus and its membrane interaction. Using this approach, it was possible to clearly show that the N-terminus is long enough to reach the surrounding membrane and that it interacts preferentially with anionic lipids (Jiang et al 2017; Lev et al 2023).

**Fig. 6:**
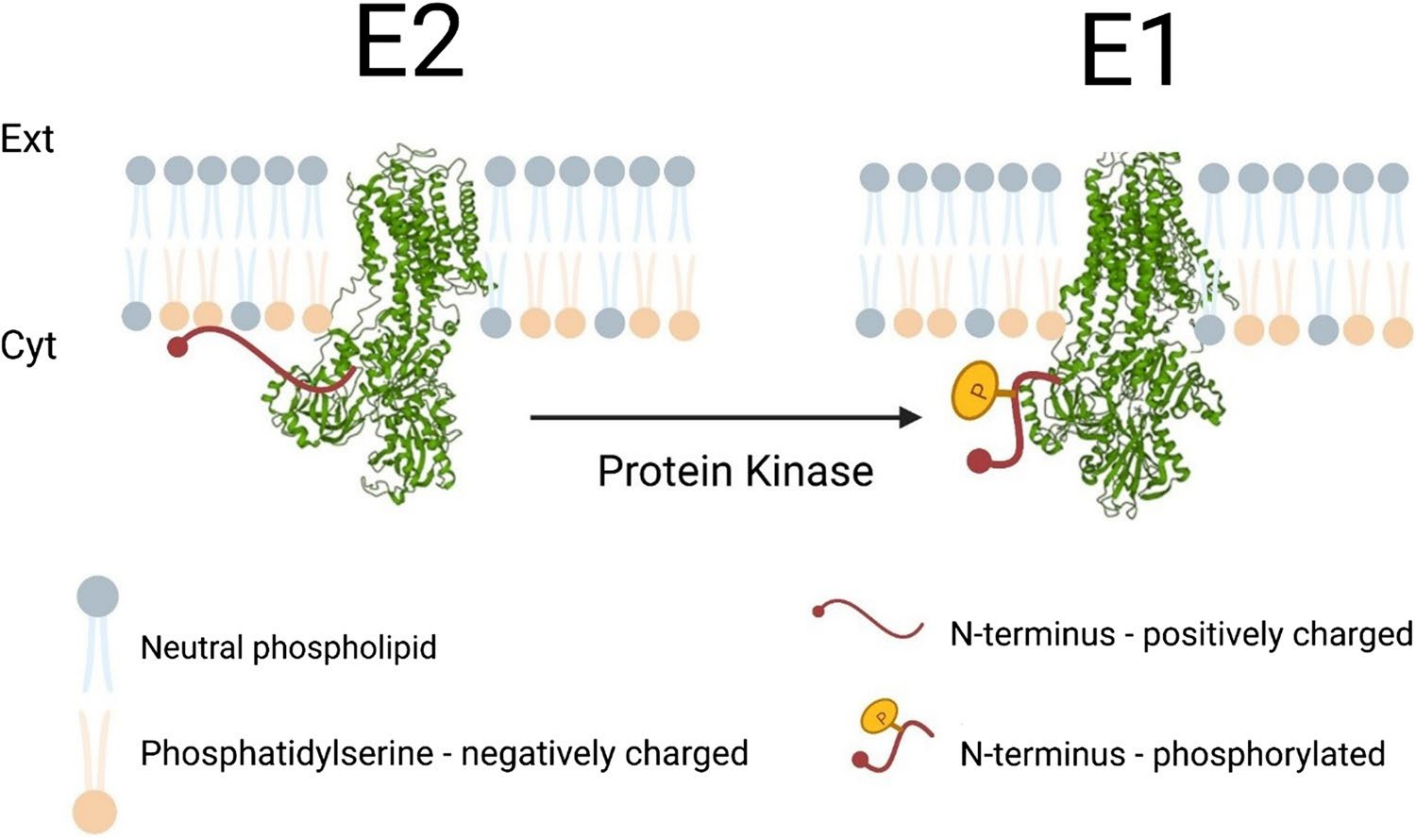
Proposed operation of the electrostatic switch mechanism in the regulation of the Na^+^,K^+^-ATPase (reproduced from Blayney et al 2023). The E2 conformation of the enzyme is stabilized via an electrostatic interaction between positively charged lysine residues on the N-terminus and the negatively charged headgroups of phosphatidylserine on the cytoplasmic surface of the surrounding membrane. Phosphorylation of hydroxyl groups of conserved serine and tyrosine residues of the N-terminus by protein kinases introduces negative charges onto the N-terminus, weakening its electrostatic interaction with the membrane and causing its release from the membrane. This destabilizes the E2 conformation of the protein and facilitates its conformational into the E1 state. The E2 and E1 structures shown in the figure were derived from published crystal structures PDB 3B8E in the case of the E2 state (Morth et al 2007) and PDB 3WGU in the case of the E1 state (Kanai et al 2013)

A schematic diagram of how the electrostatic switch mechanism could function for the Na^+^,K^+^-ATPase is shown in Fig. 6. This shows phosphorylation of the N-terminus by a protein kinase, which increases the negative charge on the N-terminus, breaking its interaction with the negatively charged membrane surface and forcing it off the membrane. This then favors the enzyme’s E1 conformational state over the E2 state. Based on in vitro experiments in the absence of protein kinases (Garcia et al 2017; Jiang et al 2017), it seems that movement of the N-terminus on and off the membrane is part of the normal ion-transporting reaction cycle of the Na^+^,K^+^-ATPase. The role of the electrostatic switch caused by kinase phosphorylation is to change the relative energies of different conformational states. Of course, if the energy of a particular conformational state is lowered to such a degree that it can no longer overcome the activation energy to proceed to the next conformational state in the enzyme’s reaction cycle, this would cause an inhibition. This could then be an effective mechanism for regulating the protein’s ion-pumping activity. To reverse the phosphorylation, a regulatory phosphatase would be required, but the author is not aware of any research on the specific phosphatase that fulfils this role.

The Na^+^,K^+^-ATPase and the gastric H^+^,K^+^-ATPase belong to the P2C subfamily of P-type ATPases. Another family of P-type ATPases in which polybasic clusters are present is the P4-ATPase family (Andersen et al 2016), which transport phospholipids such as PS, PE, and PC across the plasma membrane and are responsible for maintaining the transverse lipid asymmetry of the membrane. Those that transport lipids across the membrane from the extracellular leaflet to the cytoplasmic leaflet are also known as flippases. One particular flippase, the phospholipid-transporting ATPase VA, which is encoded by the gene ATP10A and which flips PC across the plasma membrane, has in many species distinct polybasic amino acid residue clusters in its cytoplasmic N-terminus, reminiscent of those of the Na^+^,K^+^-ATPase and the H^+^,K^+^-ATPase. For example, in *Homo sapiens*, ATPase VA residues 15–19 and 48–52 (numbering excluding the initiation methionine) are arginine (R). In *Gulo gulo luscus*, the wolverine, there are even eight arginine residues in a row, from 15–22. The prevalence of arginine residues is interesting, because this is different from the Na^+^,K^+^- and H^+^,K^+^-ATPases, where lysine is the dominant basic residue in their N-termini. As mentioned earlier, based on its chemical structure and charge delocalization, arginine would be expected to cause greater membrane penetration than lysine. Another interesting difference between the sequence of the ATPase VA and the Na^+^,K^+^- and H^+^,K^+^-ATPases is that the ATPase VA does not appear to have clearly conserved serine or tyrosine residues which could be targets for protein kinases. This would seem to suggest that the ATPase VA N-terminus might permanently bind to the membrane. This would be consistent with recent results of Okamoto et al. who presented evidence that the N- or C-terminal regions of P4-ATPases determine their cellular localization (Okamoto et al 2020). Thus, the arginine clusters of ATPase VA may be involved in the intracellular trafficking of the enzyme as a way of ensuring that the protein is inserted into the plasma membrane with its high level of negatively charged PS molecules on the cytoplasmic surface.

### Neurotransmitter transporters

There is also evidence that electrostatic interactions between polybasic clusters on the N-termini of neurotransmitter transporters and negatively charged lipid headgroups may regulate their function. The human dopamine transporter (hDAT) has lysine residues in positions 3 and 5, and potential sites for phosphorylation at serines in positions 2 and 4. Hamilton et al. (Hamilton et al 2014) showed that the lysine residues interact with negatively charged lipids in the surrounding membrane, but in this case with phosphatidylinositol 4,5-bisphosphate (PIP_2_), although interaction with PS cannot be excluded. They found that mutating the lysine residues to uncharged amino acid residues reduced both the interaction with the membrane and amphetamine-induced dopamine efflux by the transporter. In vivo, they found functional effects of the mutations, evidenced by changes in locomotion of *Drosophila melanogaster*. They suggested that the mechanism of PIP_2_ regulation that they identified for hDAT could also be operative in other neurotransmitter transporters, i.e., the human serotonin transporter (hSERT), the norepinephrine transporter (hNET), and the vesicular monoamine transporter 2 (hVMAT2). They based this suggestion on the fact that the sequences of these transporters also have polybasic clusters in their N-termini.

A region of strong positive charge on the cytoplasmic side of SERT due to basic amino acid residues has also been recognized by Buchmayer et al. (2013), who in addition reported reduced amphetamine-induced currents on reduction of the level of PIP_2_ in the membrane. This supports the idea that electrostatic interactions between basic amino acid residues and PIP_2_ within the membrane play an important role in the regulation of SERT. Anderluh et al. (2017) have suggested that PIP_2_ interactions with the protein’s positively charged patch stabilizes an oligomeric structure of the transporter within the plasma membrane.

### Ion channels

So far we have discussed pumps and transporters, but there is clear evidence that electrostatic interactions of polybasic protein segments with the cytoplasmic surface of the plasma membrane also regulate a number of different types of ion channels. One example is the bacterial inward rectifier K^+^ (Kir) channels (Ptak et al 2005). Some of these channels possess an RCK (regulates conductance of K^+^) domain containing many positively charged lysine residues which promote interaction with negatively charged lipid headgroups in the membrane. Experiments using purified Kir channels reconstituted into lipid vesicles showed that the interaction of the RCK domain with the membrane decreased on increasing the ionic strength and on decreasing the anionic lipid ratio of the membrane (Ptak et al 2005). Both these results are consistent with an electrostatic interaction of the RCK domain with the membrane.

Another class of channels where electrostatic interactions with the membrane play an important role in their regulation is the eukaryotic transient receptor potential (TRP) channels, a family of nonselective cation channels. The mammalian TRP vanilloid (TRPV) subfamily has long intrinsically disordered regions at their N-terminus. Of all the members of the subfamily, TRPV4 has the longest disordered region of 130–150 amino acid residues depending on the species (Goretzki et al 2021, 2022). Three-dimensional structures of some TRPV4 channels have been solved, but as in the case of the Na^+^,K^+^- and H^+^,K^+^-ATPases, discussed earlier, the intrinsically disordered N-termini were either deleted prior to structure determination or are not resolved in the structure (Deng et al 2018). The N-terminal tail contains basic residues, both lysine and arginine, which bind to PIP_2_ on the cytoplasmic face of the plasma membrane (Garcia-Elias et al 2013; Goretzki et al 2023). When the N-terminal tail is bound to PIP_2_, it is in an extended conformation which allows channel activation by hypotonicity and heat, but when it is released from PIP_2_, it adopts a compact conformation which prevents channel activation (Garcia-Elias et al 2013; Goretzki et al 2023).

An electrostatic switch mechanism has also been postulated to be involved in the regulation of epithelial sodium channels (ENaC), which are responsible for the reabsorption of Na^+^ ions in epithelial cells, particularly in the kidney, and play an important role in determining blood pressure. It has been hypothesized that ENaC is regulated by an interaction of positive charges of basic residues on the N-terminus of one or more of the channel’s subunits with anionic phospholipids in the cytoplasmic leaflet of the plasma membrane (Kleyman and Eaton 2020). In particular, PIP_2_ has been found to increase the open probability of the channel. However, neither PIP_2_ nor ENaC is present in high concentrations in the plasma membrane. The question then is, how do they find one another? A solution to this problem has been proposed by Kleyman and Eaton (2020). They have suggested that the peripheral membrane protein MARCKS concentrates PIP_2_ into localized domains within the membrane by interacting with PIP_2_ via its polybasic lysine clusters of its N-terminus and then delivers PIP_2_ to ENaC. As described earlier in the section on peripheral membrane proteins, interaction of MARCKS with the cytoplasmic membrane surface is regulated by an electrostatic switch mechanism involving phosphorylation of serine on the MARCKS N-terminus by PKC. In this way, ENaC would, via MARCKS, also indirectly be regulated by an electrostatic switch mechanism.

## Conclusion

In this review a lipid-protein interaction has been discussed which cannot be classified as either general or specific. That is, protein function is not modulated indirectly via a change in the membrane’s physical properties (general), nor do lipid molecules bind to specific sites within the protein. In the nonspecific lipid-protein interaction presented here, the lipid molecules are situated within the membrane and interact directly with the protein via a nonspecific electrostatic interaction between positively charged polybasic regions (including lysine and arginine residues) on intrinsically disordered N- or C-termini of the protein and anionic lipid headgroups (PS and PI) on the cytoplasmic surface of the membrane. This type of interaction is not rare. It is found in both peripheral and integral membrane proteins, and, in the case of integral membrane proteins, in primary active transporters (ion and lipid pumps), secondary active transporters, and ion channels. In both peripheral and integral membrane proteins, the interaction can be modulated by electrostatic switch mechanisms whereby the charge on the protein’s terminal tail or on the membrane surface is changed. The charge on the protein’s terminus can be reduced by regulatory phosphorylation by protein kinases. The charge on the membrane can be reduced by translocation of negatively charged lipids to the extracellular membrane leaflet or by neutralization of the charge by the binding of divalent metal ions, such as Ca^2+^.

An important feature of this mechanism of controlling membrane protein localization and activity is that it is dependent on the transverse lipid asymmetry of the plasma membrane, with negatively charged lipids concentrated on the cytoplasmic leaflet of the plasma membrane. This emphasizes the importance of lipid asymmetry and lipid flippase activity for cell physiology. In effect, all the membrane processes described in this review are dependent on the plasma membrane’s lipid asymmetry and are all in principle capable of being regulated by flippases.

Because the N- and C-termini of proteins involved in this type of regulation are intrinsically disordered, they cannot be resolved via cryo-electron-microscopy or X-ray crystallography. Therefore, they have often been overlooked in the past. It is likely that many more electrostatic switches await discovery.

## Data Availability

Not applicable.
